# Effect of Steel Slag on Hydration Kinetics and Rheological Properties of Alkali-Activated Slag Materials: A Comparative Study with Fly Ash

**DOI:** 10.3390/ma17102260

**Published:** 2024-05-10

**Authors:** Fanghui Han, Ziqin Zhu, Hongbo Zhang, Yuchen Li, Ting Fu

**Affiliations:** 1 Beijing Key Laboratory of Urban Underground Space Engineering, School of Civil and Resource Engineering, University of Science and Technology Beijing, Beijing 100083, China; 2 Research Institute of Urbanization and Urban Safety, University of Science and Technology Beijing, Beijing 100083, China; 3 China Construction Science and Technology Group Co., Ltd., Beijing 100070, China

**Keywords:** alkali-activated slag paste, steel slag, fly ash, hydration heat, rheological properties

## Abstract

The effects of steel slag (SS) and fly ash (FA) on hydration heat, fluidity, setting time and rheological properties of alkali-activated slag (AAS) pastes with different silicate modulus (Ms) values were comparatively investigated. The results show that the incorporation of SS shortens the induction period, increases the cumulative hydration heat, improves the initial fluidity and decreases the setting time at low Ms, but the opposite trend is found at high Ms. FA significantly retards the reaction, reduces the hydration heat, increases the fluidity and prolongs the setting time. The addition of SS or FA reduces the yield stress and plastic viscosity of AAS paste. SS improves the rheological properties of AAS paste more significantly than that of FA at high Ms. The yield stress and plastic viscosity of AAS paste with SS or FA rise with the increasing Ms and decline with the increasing water/binder (w/b) ratio.

## 1. Introduction

Engineering construction has a huge demand for Portland cement at present. Although the production equipment and technology of Portland cement have been greatly improved, the production process still consumes considerable resources and energy. To reduce the output of cement, it is necessary to find green materials that can replace or partially replace cement [1]. Alkali-activated materials (AAMs) are formed by the reaction of silicate-aluminate solid raw materials with pozzolanic activity or potential hydraulicity with alkaline activators [2]. Due to the abundant resources of ground granulated blast furnace slag (GGBS) and its relatively high activity, slag has been extensively and deeply analyzed. In addition, the properties of industrial solid wastes such as fly ash (FA), steel slag (SS) and nickel slag under alkaline conditions are also explored. AAMs have the advantages of a rich source of raw materials [3], high strength [4], low production cost [5], green and environmental protection [6], good durability [7,8,9,10,11] and excellent performance of toxic heavy metal ion solidification [12].

Although the mechanical properties and durability of alkali-activated slag (AAS) binder are excellent, the AAS binder has poor workability [13,14,15,16]. The fluidity is greatly lost in a very short period of time, and the fast setting and hardening are observed [17,18,19]. At present, many researchers use FA to improve the workability of AAS. Adding FA to AAS can obtain greater fluidity and a longer setting time, which is beneficial to the normal construction. The addition of the larger amount of FA leads to the longer the setting time [20]. The type of activator also affects the fresh performance of AAMs. The sodium silicate solution has a significant effect on the viscosity of the alkali-activated slag–fly ash (AASF) composite binder. When the Ms increased from 1.0 to 1.5, the plastic viscosity of the AASF paste doubled. The increase in slag content improved the surface interaction between particles, thereby enhancing the yield stress and plastic viscosity [21]. However, Sun et al. [22] claimed that, dissimilar to the effect of increasing the w/b ratio and SiO_2_/Na_2_O ratio on workability, a larger amount of slag and alkali could reduce the workability of AASF paste. In addition, temperature also enhanced the yield stress and apparent viscosity of AASF paste. The dissolution rate of FA was accelerated when the temperature was higher than 65 °C; thus, the precipitation of hydration products increased [23].

The production of slag is accompanied by the production of SS during the steel and iron production process. Hence, slag and SS are associated solid waste. Slag is a kind of pozzolanic materials, but SS contains minerals of C_2_S (2CaO·SiO_2_), C_3_S (3CaO·SiO_2_), C_2_F (2CaO·Fe_2_O_3_) and C_4_AF (4CaO·Al_2_O_3_·Fe_2_O_3_), resulting in it having its own hydraulic properties. The Ca(OH)_2_ generated via the hydration of SS can promote the reaction of slag. However, FA is the solid waste of power plants. Slag and FA are not produced in the same plant. As a result, the SS and slag have a natural location advantage, which is favorable to product AAMs. The reactivities of SS and FA are lower than that of slag. SS has the improving effect on the fluidity of Portland cement paste [24]. If the SS, like FA, can improve the workability of AAS paste, it will be well applied in AAS material. More importantly, the Ca(OH)_2_ produced by the hydration of SS can further accelerate the reaction of slag. Nevertheless, few researchers have systematically studied the properties of AAS material containing SS. The reaction process and the products of alkali-activated SS binder were similar to those of Portland cement, but the alkali-activated SS binder showed a shorter induction period, smaller peak value and lower total hydration heat [25]. The hydration heat of the alkali-activated SS binder presented four hydration stages at 60 °C: dissolution, acceleration, deceleration and stabilization. Two hydration exothermic peaks were observed in the exothermic rate curve. The first peak was correlated with the wetting and dissolution of SS particles, while the second peak was attributed to the reaction of SS [26]. The incorporation of 50% SS prolonged the setting time and increased the fluidity of alkali-activated slag–steel slag (AASS) paste [27]. Na_2_SO_4_ could accelerate the early reaction of concrete containing 40% SS due to the activation effect of the activator on SS, and the workability of the concrete decreased with the increasing Na_2_SO_4_ content [28].

The rheological properties of fresh pastes have a major impact on many aspects of concrete, such as pumpability, ease of pouring and consolidation, segregation resistance, strength and durability [29,30,31]. However, few published studies explored the workability of AASF paste. More importantly, there are few studies focusing on the exothermic hydration process of AASF paste with different Ms values. Therefore, in this paper, 50% slag was replaced by SS in the AAS paste. The influences of Ms on the hydration heat, fluidity, setting time and rheological properties of AASS were investigated. The effects of SS on the hydration kinetics and rheological properties of AAS paste were comparatively analyzed and discussed with those of AAS paste containing the same amount of FA, and the influence mechanisms were also given. 

## 2. Materials and Methods

### 2.1. Raw Materials

**Figure 1:**
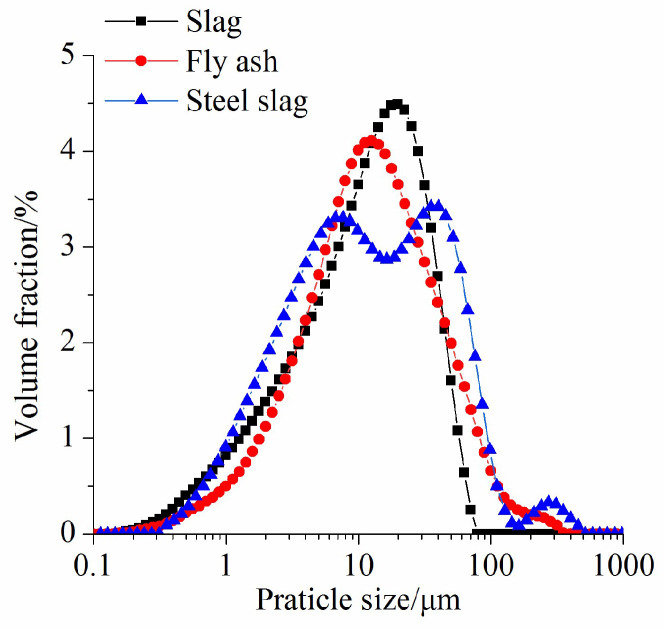
Particle size distribution curves of raw materials.

S95 GGBS, Class I FA and basic oxygen furnace SS produced by Shandong JinTaicheng Construction Technology Co., Ltd, Linyi, China, were used in this study. Table 1 shows the chemical compositions of the three raw materials. The main compositions of the slag are SiO_2_, CaO, Al_2_O_3_ and MgO. The main compositions of FA are SiO_2_ and Al_2_O_3_, of which the SiO_2_ content is the highest, up to 57.6%, but the CaO content is the lowest, i.e., only 3.87%. The main compositions of SS are CaO, Fe_2_O_3_ and SiO_2_, of which SiO_2_ content is the lowest among the three materials, but the contents of CaO and Fe_2_O_3_ are relatively high. Figure 1 presents the particle size distribution curves of the raw materials. It is apparent that the particle size of slag is the smallest and the large particles exist in SS. Owing to the existence of the RO phase (CaO-FeO-MgO-MnO solid solution) in the SS, large particles (>100 μm) are found. 

The sodium silicate solution was used as activator. The Ms of the sodium silicate solution was adjusted by sodium hydroxide. Sodium silicate solution was produced by Shandong Yousuo Chemical Technology Co., Ltd., Linyi, China. Sodium hydroxide was an analytical reagent produced by Xilong Chemical Co., Ltd., Shantou, China. Deionized water and laboratory tap water were used for the experiments of hydration heat and other experiments, respectively. Superplasticizer with a water-reducing rate of 30% produced by Jiangsu Subot New Materials Co., Ltd., Nanjing, China, was added to ensure the fluidity of the paste.

### 2.2. Mix Proportions

Due to the release of a great amount of heat during the preparation of the sodium silicate solution, the sodium silicate solutions with different Ms values were prepared 24 h in advance before the experiments. Then, 1% superplasticizer was added when the pastes were prepared with a w/b ratio of 0.4. The mix proportions of the pastes are given in Table 2, for example, where GS1 and GS1′ represent the sample with 0.5 Ms at 0.4 and 0.5 w/b ratio, respectively.

### 2.3. Test Methods

The pastes were prepared according to Table 2 for the tests of hydration heat, fluidity, setting time and rheological properties. To ensure the accuracy of the data, the experiments of fluidity, setting time and rheology of AAS paste containing SS or FA were repeated 3 times for every mixing ratio of experiment.

The hydration heat of the paste was tested with a TAM Air isothermal calorimeter with eight channels. The heat evolution rate and cumulative hydration heat of the samples were measured continuously for 72 h under 25 °C.

The fluidity of AAM paste was carried out conforming to the Chinese National Standard GB/T8077-2012. The prepared paste was poured into a small slump cone, and the slump cone was slowly lifted vertically. The maximum diameters of the fluidity in two mutually perpendicular directions were measured with a ruler after 30 s. The fluidity was the average value. To ensure the accuracy of the data, the above experiment should be repeated 3 times.

The setting time of the prepared paste was determined in light of the Chinese National Standard GB/T1346-2011. The initial setting needle touched the surface of the paste and suddenly came loose. When the needle sank to 4 ± 1 mm from the bottom plate, the paste reached the initial setting state. When the annular attachment of the final setting needle could not leave traces on the surface of the test sample, the paste reached the final setting time. 

The rheological curves of the pastes were measured 5 min, 60 min and 120 min after their preparation by means of a Brookfield RV-III rheometer. The paste was cured in standard curing equipment before the rheological test. The paste was stirred at the same speed for 1 min before measurement. The distance between the rotor of the rheometer and both the bottom and the upper surface of the paste was 2 cm. To obtain accurate test results, the rotor of the rheometer was kept right in the middle of the paste. The rheological test procedure was the same as that in the literature [32]. The rheological parameters of the pastes were obtained by fitting the rheological curve with the Herschel–Bulkley (H-B) model [33]. The H-B rheological model is as follows:

(1) $$\tau =\tau_{0}+K\gamma^{n}$$

where *τ* and *τ*_0_ represent the shear stress (Pa) and yield stress (Pa), respectively. *K* and *n* are the consistency coefficient (Pa·s^n^) and rheological index, respectively. When *n* = 1, the fluid shows Bingham rheological behavior; when *n* < 1, the fluid shows shear thinning rheological behavior; and when *n* > 1, the fluid shows shear thickening rheological behavior. In addition, *γ* is the shear rate (s^−1^).

The plastic viscosity, *μ* (Pa·s), was obtained according to Equations (2) and (3) [34,35,36]:

(2) $$\mu =\frac{3K}{n+2}{\gamma_{\max}}^{n-1}$$

(3) $$\gamma_{\max}=100\;s^{-1}$$

where *γ*_max_ is the maximum shear rate (s^−1^).

## 3. Results and Discussion

### 3.1. Hydration Heat

**Figure 2:**
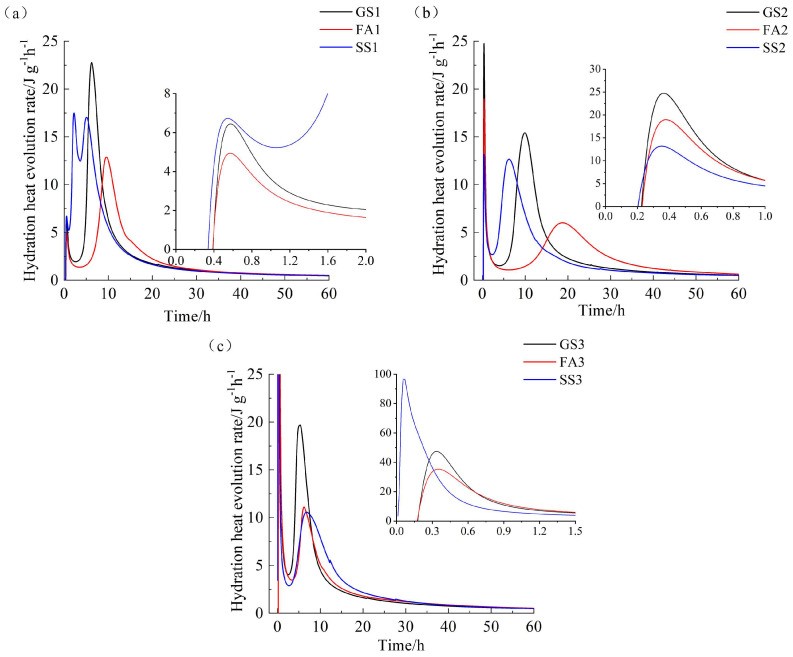
Hydration heat evolution rate of AAS containing FA or SS with different Ms values: (**a**) 0.5 Ms, (**b**) 1.0 Ms and (**c**) 1.5 Ms.

Figure 2 illustrates the hydration heat evolution rates of the three materials with different Ms values. The exothermic rate curve of AAS and AAS containing SS or FA contains five periods: the initial period, induction period, acceleration period, deceleration period and steady period [37]. Two exothermic peaks appear in the exothermic rate curves of GS1 and FA1 (Figure 2a). However, three exothermic peaks appear on the exothermic rate curve of SS1, which is obviously different from FA1. The first exothermic peaks of all samples are observed when the precursors contact the sodium silicate solution. The first exothermic peak values of the three samples are low. The second exothermic peak is caused by the formation of a large amount of C-(A)-S-H gel due to the reaction of Ca^2+^ dissolved from precursors with SiO_4_^4−^ in sodium silicate solution. The addition of FA prolongs the induction period, delays the appearance of the second exothermic peak and reduces the peak value. This is consistent with the research results of Sun et al. [38]. What is interesting is that the addition of SS presents a different change rule. The second and third exothermic peaks of SS1 both appear before the second exothermic peak of GS1. The second and third exothermic peaks of SS1 are generated by the reactions of SS and slag, respectively. The dissolution rate of SS is faster at a low Ms [39]. Meanwhile, the SS and slag mutually promote the reaction, and then the appearing times of the two exothermic peaks are anticipated. As shown in Figure 2b, the appearing time of the second exothermic peak of FA2 is delayed, and the peak value is reduced more obviously at 1.0 Ms. However, SS2 has only two exothermic peaks. The appearing time of the second exothermic peak of SS2 is still anticipated, but the peak value decreased compared to GS2. The dissolution rate of SS is still fast at 1.0 Ms, which is attributed to the higher content of CaO of SS. The dissolved Ca^2+^ from SS can react with the SiO_4_^4−^ provided by the alkali activator [40]. However, compared with slag, the activity of SS is relatively lower [27]. Thus, SS2 exhibits a lower exothermic peak value. The excitation effect of the sodium silicate solution becomes poorer at 1.0 Ms due to the reduction in the pH value. However, it is equivalent to providing more silicate ions when the Ms increases. Silicate ions can participate in the reaction, thereby promoting the reaction. The effect of alkalinity reduction on the early reaction of SS2 is smaller than the promotion effect of silicate ions. The first exothermic peak value of SS3 is significantly higher than that of GS3 and FA3 (Figure 2c). The second exothermic peak values of SS3 and FA3 are significantly lower than that of GS3. The addition of SS retards the AAS reaction at 1.5 Ms. The ending time of the induction period and the second exothermic peak value of SS3 are almost the same as those of FA3.

The activator provides more silicate ions for the hydration process when the Ms increases, thereby affecting the formation and hydration process of hydration products. It can be seen from Figure 2a–c that, for AAS and AASF, the rise of Ms leads to the increase in the first exothermic peak and the first decrease and then increase in the second exothermic peak. Meanwhile, Ms also affects the occurrence time of the second exothermic peak. The increase in Ms first extends and then shortens the appearance of the second exothermic rate peak. The second exothermic peak value reduces due to the decrease in alkalinity at 1.0 Ms. However, the reaction is accelerated, and the induction period is shortened due to the increase in SiO_4_^4−^ with a further increase in Ms. The higher SiO_4_^4−^ concentration and the lower pH value promote the dissolution of calcium, which accelerates the reaction process. The effect of Ms on the exothermic rate of AASS is different from that of AAS and AASF. An increase in Ms decreases the second exothermic peak value of AASS and delays its appearing time. This is mainly due to the low dissolution rate of SS at high Ms. 

**Figure 3:**
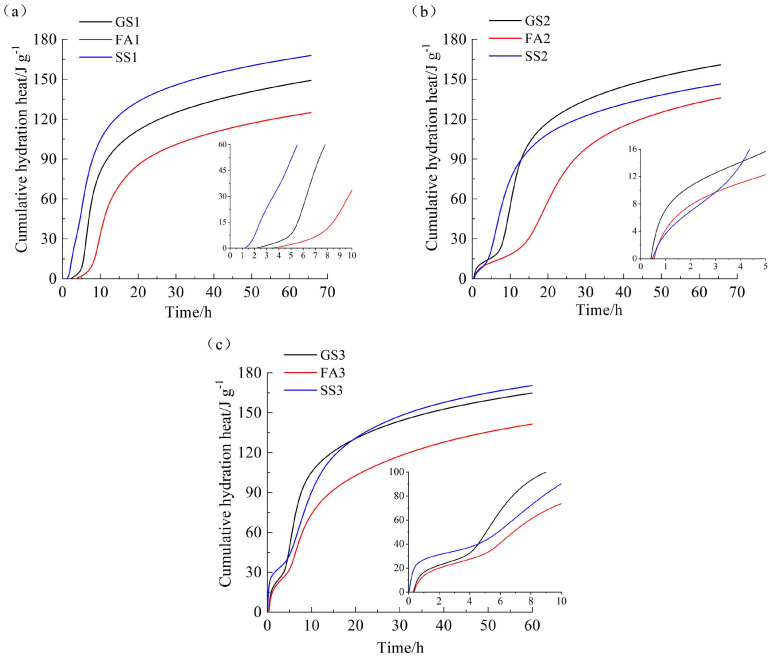
Hydration heat evolution rate of AAS containing FA or SS with different Ms values: (**a**) 0.5 Ms, (**b**) 1.0 Ms and (**c**) 1.5 Ms.

Figure 3 shows the cumulative hydration heat of AASS and AASF activated by sodium silicate with different Ms values. The cumulative hydration heat of FA1 is smaller than that of GS1 (Figure 3a), which is consistent with the research results of Sun et al. [38]. However, the opposite trend is found for SS1. Because the dissolution rate of SS is fast at low Ms, the hydration heat dramatically increases due to the mutual promotion of the reaction between SS and slag. SS2 presents greater cumulative hydration heat within 14 h, but a lower cumulative hydration heat within 72 h compared to GS2 (Figure 3b). The dissolution rate of SS at 1.0 Ms is lower than that at 0.5 Ms, which leads to lower cumulative hydration heat at 1.0 Ms. However, the cumulative hydration heat of SS2 is obviously higher than that of FA2. The cumulative hydration heat of SS3 within 5 h is greater than that of GS3. This is related to the high dissolution heat of SS3 at 1.5 Ms (Figure 2c). As a result of the rapid reaction of slag at 1.5 Ms (Figure 2c), the cumulative hydration heat of SS3 within 20 h is lower than that of GS3. It is worth noting that the cumulative hydration heat of SS3 within 72 h is slightly higher than that of GS3. More hydration products generated by the early reaction of slag are attached to the surface of unhydrated slag particles, thus limiting the further reaction of slag. In addition, the increase in silicate ions compensates for the reduction in the dissolution rate of SS at a high Ms. 

It can be seen in Figure 3a–c that the cumulative hydration heat of AAS and AASF increases with the increase in Ms. This is due to the increase in SiO_4_^4−^ with the increasing Ms [41], thereby accelerating the formation of hydration products and increasing the hydration heat. Nevertheless, an increase in Ms decreases first and then increases the cumulative hydration heat of AASS. The dissolution rate of SS is fast at a low Ms. The dissolution rate decreases, and the reaction slows down, when the Ms increases. However, the increased amount of SiO_4_^4−^ at a high Ms further accelerates the reaction and releases more hydration heat from AASS. 

### 3.2. Fluidity

**Figure 4:**
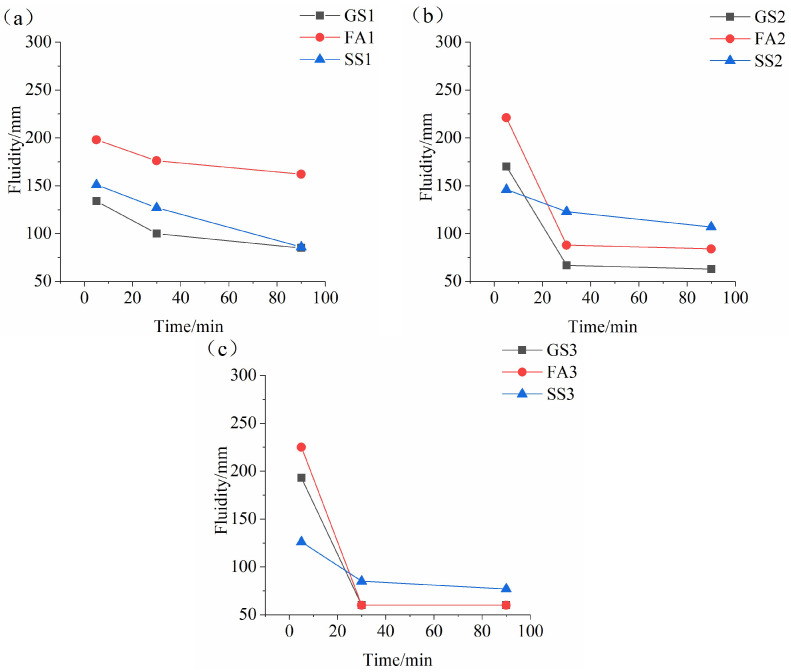
Fluidity of the AAS paste containing SS or FA with different Ms values when the w/b ratio is 0.4: (**a**) 0.5 Ms, (**b**)1.0 Ms and (**c**) 1.5 Ms.

Figure 4 displays the fluidity of the AAS paste containing SS or FA prepared with a w/b ratio of 0.4 at different Ms values. Figure 4a shows that FA or SS increases the initial fluidity of the AAS fresh paste at 0.5 Ms, especially FA. The fluidity keeps decreasing from 5 min to 90 min, and the decreasing ratio of SS1 is much greater. The main influence factors of the initial fluidity are the dissolution rate and dispersion of the material in the alkali activator solution during the dissolution period. The greater dissolution rate and better particle dispersion in the solution result in greater initial fluidity [42]. In addition, the initial fluidity is also related to the particle shape. The round particles lead to greater initial fluidity [43,44]. FA has a spherical shape and an excellent ball-bearing effect. Therefore, the addition of FA increases the initial fluidity of AAS paste. However, when the Ms is 0.5, the dissolution rate of slag is low, while the dissolution rate of SS is high. Therefore, the initial fluidity of SS1 is greater than that of GS1. The fluidity of all samples decreases at 30 min and 90 min due to the hydration reaction. SS1 and GS1 almost have the same fluidity at 90 min.

As shown in Figure 4b, the initial fluidity of FA2 is still greater than that of GS2. The initial fluidity of SS2 is smaller than that of GS2 due to the slower dissolution rate of SS. However, the variation laws of the fluidity at 30 min and 90 min are obviously different. The fluidity of GS2 is minimum, while SS2 has the maximum fluidity. The reaction rate of slag is faster due to its higher activity. The generated hydration products densify the structure and reduce the fluidity of the AAS paste. However, the reaction rate of SS2 is slower, leading to small amount of hydration products, resulting in the large fluidity at 30 min and 90 min. The fluidity of SS2 is larger than that of FA2 at 30 min and 90 min. As seen in Figure 4c, the changing regular of the initial fluidity of the three samples at 1.5 Ms is similar to that at 1.0 Ms. Since sodium silicate solution with 1.5 Ms provides a large amount of SiO_4_^4−^, which accelerates the reaction of the three samples. The fluidity of GS3 and FA3 is reduced to 60 mm at 30 min. The fluidity of SS3 is reduced to 85 mm and 77 mm at 30 min and 90 min, respectively. The results indicate that the improving effect of SS on fluidity of AAS paste is more significant than that of FA at high Ms.

It can be seen in Figure 4a–c that the initial fluidity of AAS and AASF pastes increase with increasing Ms, which is related to the fluidizing effect of sodium silicate solution [38]. This is consistent with the findings of the literature [45,46]. The vitreous network structures of slag and FA are destroyed under the activation by the sodium silicate solution. The activator with a higher Ms can provide a better coating on the surface of the precursor particles. The fluidity of the coating solution increases until the coating solution reaches a certain thickness, and the lubrication effect is fully satisfied with increasing Ms [22]. 

However, because Ca^2+^, Si^4+^ and Al^3+^ that dissolved from slag and FA can further react with SiO_4_^4−^ and OH^−^ provided by the activator to form C(N)-A-S-H gel. The fluidity of the AAS paste and AASF paste decrease sharply with increasing Ms at 30 min and 90 min. Note that the initial fluidity of AASS paste decreases with increasing Ms. The main reason is that the dissolution of the SS particles decreases with increasing Ms. SS particles cannot be well dispersed in the solution. The viscosity of the paste increases, and the fluidity becomes poor. The degree of reduction in fluidity is also lower at 30 min and 90 min.

**Figure 5:**
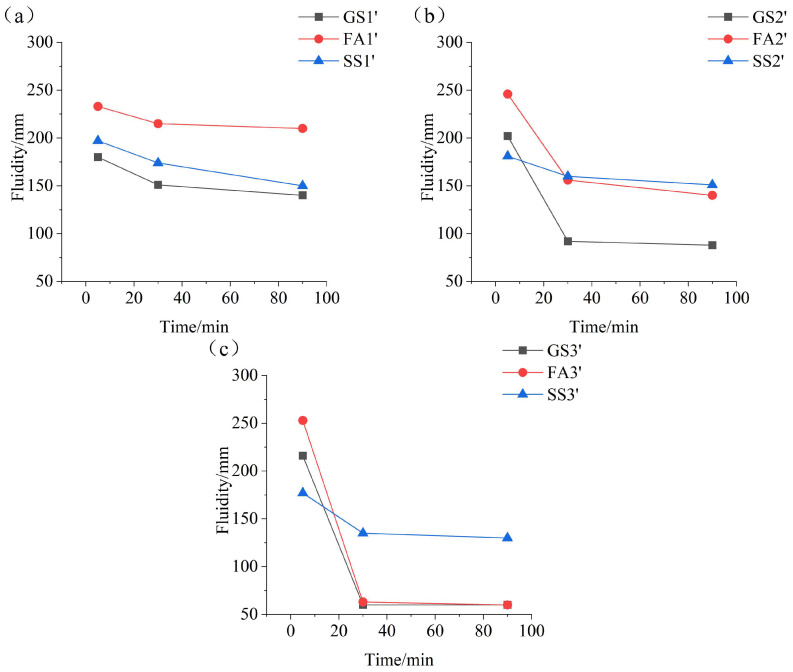
Fluidity of the AAS paste containing SS or FA with different Ms values when the w/b ratio is 0.5: (**a**) 0.5 Ms, (**b**) 1.0 Ms and (**c**) 1.5 Ms.

Figure 5 gives the fluidity of the AAS paste containing SS or FA with different Ms values when the w/b ratio is 0.5. The initial fluidity of FA1’ is obviously higher than that of GS1’ (Figure 5a). However, SS1’ has slightly higher initial fluidity than GS1’. The fluidity of the paste decreases with the increasing time, but the fluidity of FA1’ and SS1’ is always higher than that of GS1’. The addition of FA still increases the initial fluidity of AAS paste at 1.0 Ms (Figure 5b). However, the initial fluidity of SS2’ is slightly lower than that of GS2’. This is related to the slow dissolution rate of SS particles at 1.0 Ms. Due to the high reaction rates of slag and FA, the fluidity of GS2’ and FA2’ decrease obviously at 30 min and 90 min. The fluidity of FA2’ is already lower than that of SS2’ at 30 min and 90 min. When the Ms increases to 1.5 Ms, the initial fluidity of FA3’ is still higher than that of GS3’ (Figure 5c). However, the addition of SS obviously decreases the initial fluidity of AAS paste. Note that the fluidity of GS3’ and FA3’ decreases more obviously at 30 min. When the Ms is 1.5, the fluidity of the three samples has the same variation rule at the two studied w/b ratios (Figure 4c and Figure 5c).

As illustrated in Figure 5a–c, the influences of the Ms on the fluidity of AAS paste containing SS or FA are consistent at two studied w/b ratios. Sun et al. [47] found that an emulsion phase had been observed between slag particles, which consisted of discontinuous water droplets and continuous silicate-gel water glass as activation when water glass was used as the activator. Fine emulsions with smaller water droplets were observed when the Ms increased, which dispersed the slag particles.

It can be found in Figure 4 and Figure 5 that an increase in the w/b ratio from 0.4 to 0.5 increases the initial fluidity of the paste at the same Ms value. An increase in the amount of water leads to increasing the content of free water in the paste. In addition, the increase in water content reduces the concentration of the activator and weakens its activation effect. The concentration of particles participating in the reaction is also decreased. The reaction rate is therefore reduced.

To further compare the effects of SS and FA on the fluidity of AAS paste, the fluidity loss rate of the paste can be calculated by Equation (4).

(4) $$F_{1}=\frac{\left(F_{0}-F_{v}\right)}{F_{0}}\cdot 100\%$$

where *F*_1_ represents the fluidity loss rate (%); and *F*_0_ and *F_v_* represent the initial fluidity (5 min, mm) and the fluidity after rest time (30 min and 90 min, mm), respectively.

**Figure 6:**
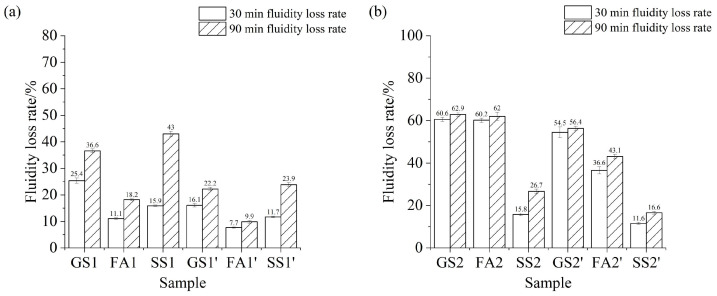

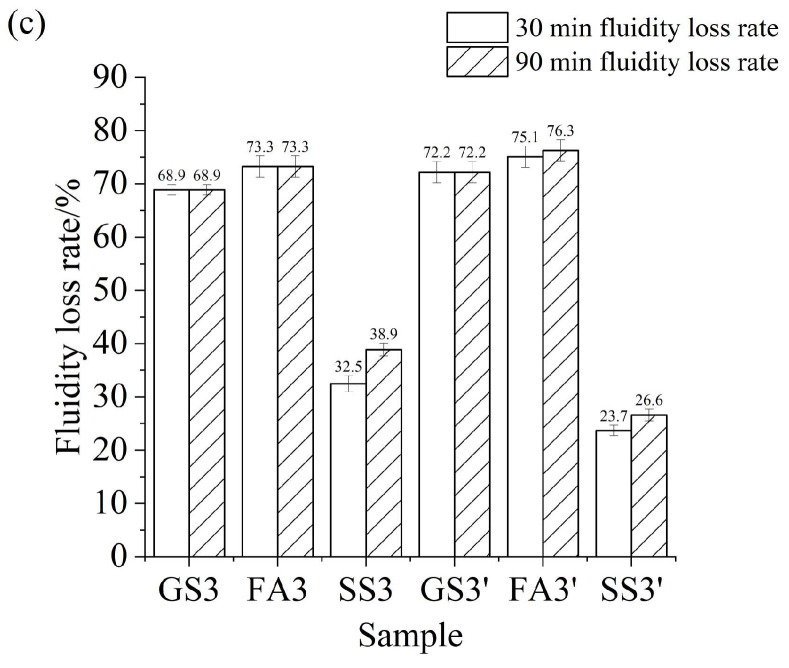
Fluidity loss rate of the AAS paste containing SS or FA with different Ms values when the w/b ratio is 0.5: (**a**) 0.5 Ms, (**b**) 1.0 Ms and (**c**) 1.5 Ms.

Figure 6 gives the fluidity loss rate of AAS paste containing SS or FA with different Ms values. Adding FA decreases the fluidity loss rates of the AAS paste with two studied w/b ratios at 30 min and 90 min when the Ms is 0.5 (Figure 6a). This result indicates that the addition of FA reduces the reaction rate of AAS. The addition of SS reduces the fluidity loss rate of AAS paste at 30 min but increases the fluidity loss rate of AAS at 90 min. It is elucidated that SS and slag react rapidly under alkali activation conditions from 30 min to 90 min at 0.5 Ms. The addition of SS or FA reduces the fluidity loss rate of AAS paste at 30 min and 90 min regardless of the w/b ratio of 0.4 or 0.5 at 1.0 Ms (Figure 6b). The fluidity loss rate of AASS paste is lower than that of AASF paste under the same conditions, indicating that the reaction rate of AASS is slower than that of AASF at 1.0 Ms. Figure 6c shows that the fluidity loss rates of AAS paste and AASF paste at 30 min are nearly equal to that at 90 min. This is due to the loss of flowability of the two samples at 30 min and 90 min at 1.5 Ms. However, AASS paste still has a low fluidity loss rate. Because the solubility of SS is low, the reaction rate is reduced at a high Ms (Figure 2c). Meanwhile, the initial fluidity of SS3 is also low. Thus, the addition of SS reduces the fluidity loss rate of AAS paste.

It can be found in Figure 6a–c that increasing the w/b ratio reduces the fluidity loss rate. The increase in water content allows the particles to be better dispersed in the dissolution. The distance between the particles is relatively increased. The concentration of reactants is reduced. The time required for the reaction is correspondingly prolonged. This phenomenon is more obvious for AASS paste. However, the fluidity loss rates of AAS paste and AASF paste at 1.5 Ms increase with the increasing w/b ratio. This is attributed to the greater initial fluidity of GS3’ and FA3’. The fluidity of GS3’ and FA3’ are almost reduced to 60 mm at 30 min. Therefore, the fluidity loss rates of GS3’ and FA3’ are higher than those of GS3 and FA3, respectively. 

It is also found that the activation effect of sodium silicate on the reaction of the slag system and the slag–fly-ash system becomes stronger with increasing Ms. However, the activation effect of sodium silicate on the slag–steel-slag system is complicated with an increasing Ms, because the solubility and the reaction degree of SS decrease with the increasing Ms. Therefore, the fluidity loss rate of AASS paste firstly decreases and then increases with the increase in Ms.

### 3.3. Setting Time

**Figure 7:**
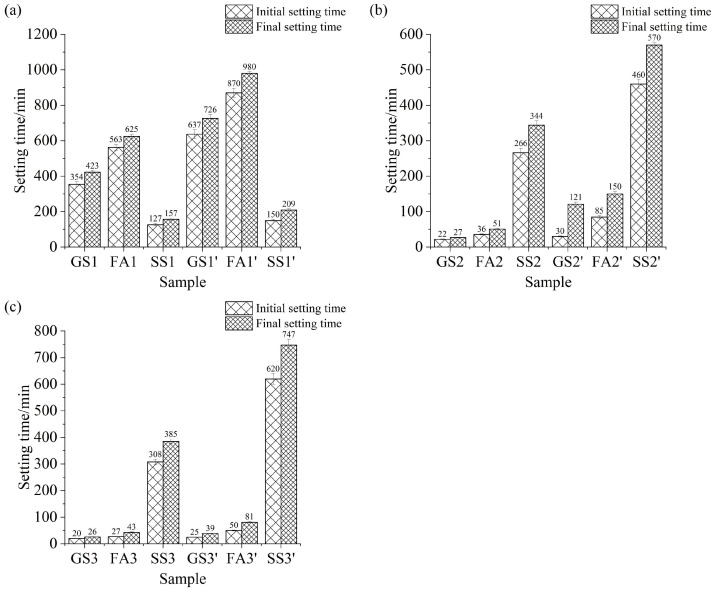
Setting time of AAS paste containing SS or FA with different Ms values: (**a**) 0.5 Ms, (**b**) 1.0 Ms and (**c**) 1.5 Ms.

Figure 7 exhibits the setting time of AASF paste and AASS paste activated by sodium silicate with different Ms values. Figure 7a shows that FA prolongs the setting time of AAS paste at 0.5 Ms. The lower activity of FA is due to the high degree of crystallization and less amorphous phase of FA. This is consistent with the results of Nedunuri et al. [20]. Meanwhile, the setting time of the AAS paste is shortened by adding SS at 0.5 Ms. As mentioned above, the pH value of the solution is higher at low Ms, which promotes the reaction of the AASS (Figure 2a). It is beneficial to the dissolution and condensation of slag and SS, and the setting time is shortened. As shown in Figure 7b, the AAS paste with a w/b ratio of 0.4 sets in a very short time. The initial and final setting times of AAS paste are 22 min and 37 min, respectively. Although the setting time is prolonged after mixing with FA, AASF paste also sets in a very short time. However, the setting time is evidently prolonged after adding SS. The decreased pH value at high Ms value decreases the reaction of SS. Therefore, the setting time is significantly longer. It is clear that the change rule of setting time at 1.5 Ms (Figure 7c) is similar to that at 1.0 Ms (Figure 7b). However, when the Ms value increases to 1.5, the setting times of the AAS paste and AASF paste are further shortened, but the setting time of the AASS paste is further prolonged. It is indicated that SS can significantly prolong the initial and final setting times of AAS paste respect to FA at a high Ms.

From Figure 7a–c, it is found that the increase in the w/b ratio extends the initial and final setting time of all investigated samples. The findings are in line with the test results of the fluidity (Figure 4 and Figure 5). In addition, it is apparent that the setting and hardening of AAS paste and AASF paste can be accelerated with increasing Ms, which lead to the shorter initial and final setting times at high Ms. However, the initial and final setting times of AASS increase with increasing Ms, which is in accordance with the test results of the fluidity (Figure 4 and Figure 5). This indicates that SS has significant retarding effect at high Ms.

### 3.4. Rheological Properties

**Figure 8:**
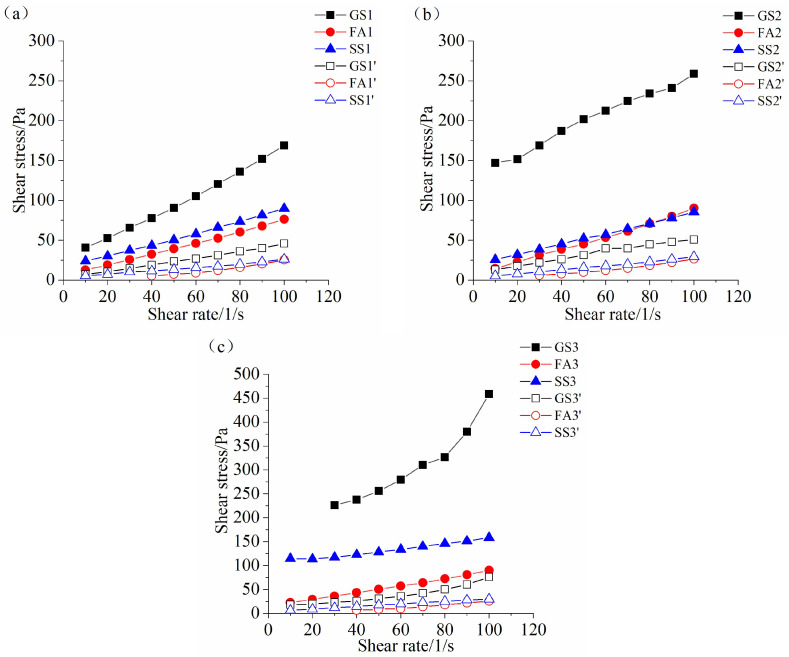
Rheological curve of the AAS paste containing SS or FA with different Ms values at 5 min: (**a**) 0.5 Ms, (**b**) 1.0 Ms and (**c**) 1.5 Ms.

**Figure 9:**
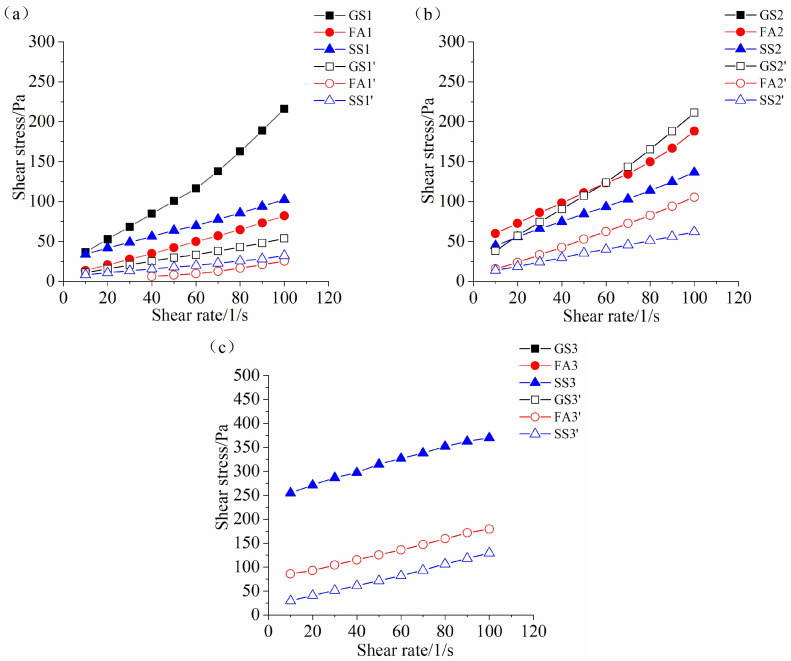
Rheological curve of the AAS paste containing SS or FA with different Ms values at 60 min: (**a**) 0.5 Ms, (**b**) 1.0 Ms and (**c**) 1.5 Ms.

**Figure 10:**
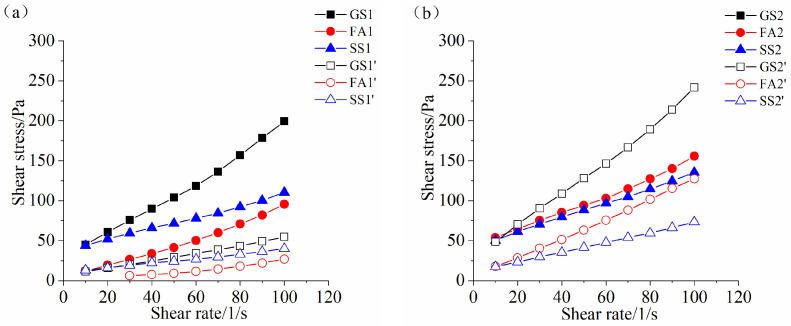

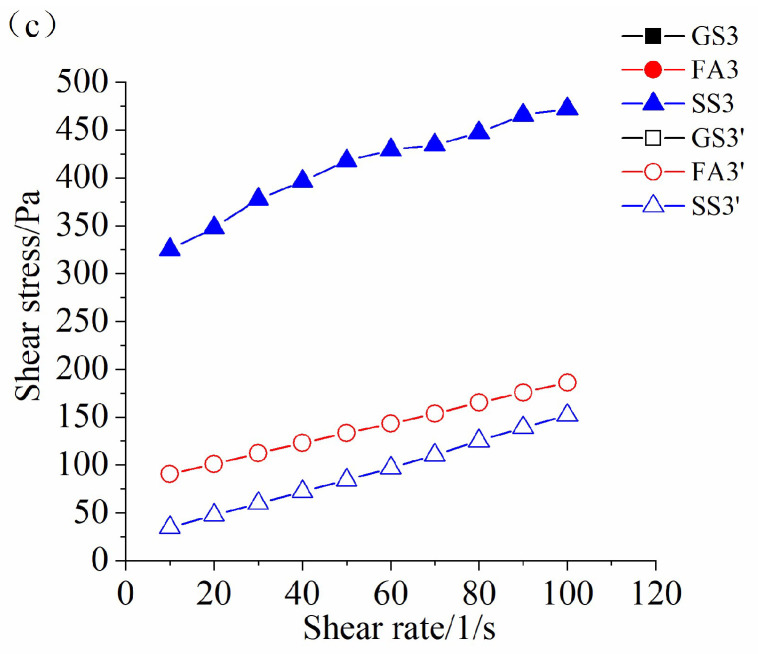
Rheological curve of the AAS paste containing SS or FA with different Ms values at 120 min: (**a**) 0.5 Ms, (**b**) 1.0 Ms and (**c**) 1.5 Ms.

Figure 8, Figure 9 and Figure 10 display the rheological curves of the AASF and AASS activated by sodium silicate with different Ms values at 5 min, 60 min and 120 min, respectively. As expected, the shear stresses of all samples increase with increasing shear rate. It is obvious that the rheological curve is nonlinear. The H-B model is used to fit the rheological curves of AASF and AASS pastes. Figure 8a shows that the addition of 50% SS or FA has the same effect, both of which decrease the shear stress of AAS paste, but the reduction effect is more obvious when FA is added. Slag has high activity under alkaline conditions. The particle size of slag is the smallest among the three materials (Figure 1). The small slag particles easily agglomerate, which reduces the dispersion rate and increases the water demand. This strengthens the structure and results in a high shear stress. The dilution effect is obvious after adding FA [48]. The spherical geometry and smooth surface of FA particles promote particle sliding, reduce the friction between particles and improve the rheology of the paste. The surface of SS particles is rougher than that of FA. SS particles absorb more water when they play a physical filling role at the early stage. Therefore, although the shear stress is reduced after adding SS at 5 min, the reduction effect of SS on the shear stress of AAS paste is smaller than that of FA. Meanwhile, it is clear that the w/b ratio also significantly affects the rheological curve of the AASS and AASF pastes. Increasing the w/b ratio also significantly decreases the shear stress of the paste, especially for AAS paste. As shown in Figure 8b,c, the shear stresses of all samples also increase nonlinearly with the increasing shear rate when Ms value increases to 1.0 and 1.5 Ms. The effects of FA, SS and w/b ratio on the rheological curve of AAS paste at 1.0 and 1.5 Ms are almost the same as those at 0.5 Ms. Note that the shear stress of the AAS paste prepared with 1.5 Ms increases significantly at a w/b ratio of 0.4.

When the w/b ratio is 0.4, an increase in Ms sharply increases the shear stress of AAS fresh paste but slightly increases the shear stress of the AASF fresh paste. As explained above, it is attributed to the dilution effect and ball-bearing effect of FA. The shear stress of the AASS fresh paste does not change much when Ms increases from 0.5 to 1.0. However, the shear stress of the AASS fresh paste increases sharply when Ms increases from 1.0 to 1.5, which is related to the low dissolution rate of SS and the high viscosity of the sodium silicate solution at high Ms. The shear stresses of all samples increase slightly with increasing Ms at w/b ratio of 0.5, which indicates that the influence of Ms on the shear stress of AASS and AASF fresh pastes is limited at high w/b ratio. 

The variation law of the rheological curves of all samples prepared with 0.5 Ms at 60 min is similar to that at 5 min (Figure 8a and Figure 9a). Note that the rheological properties of AAS paste with 1.0 Ms cannot be measured at 60 min at w/b ratio of 0.4 (Figure 9b), which is due to the fast reaction of slag and the setting of AAS paste at 60 min (Figure 7b). The shear stress of SS2 is smaller than that of FA2 (Figure 9b), which is different from the change rule at 5 min. The reaction rate of SS2 is slower (Figure 2b), and the dense structures cannot be formed. Hence, the shear stress of SS2 at 60 min is small. This is in accordance with the results of the fluidity (Figure 4b) and the setting time (Figure 7b). When the w/b ratio is 0.5, the shear stress of GS2’ is the highest, followed by that of FA2’ and SS2’ (Figure 9b), which is the same as the change law at w/b ratio of 0.4. Samples GS3, FA3 and GS3’ are set at 60 min (Figure 7c). Thus, the rheological curves of these three samples cannot be obtained at 60 min (Figure 9c). The shear stress of SS3 is relatively large, and the shear stress of SS3’ is significantly lower than that of FA3’ at 60 min.

When the rest time increases to 120 min, the change rules of the shear stresses of all samples with different Ms values are almost the same as those at 60 min (Figure 9 and Figure 10). However, the shear stress is much higher after a longer rest time. An increase in Ms from 0.5 to 1.0 or 1.5 significantly increases the shear stress of AASS and AASF pastes at 120 min, especially for GS2’ and SS3 (Figure 10b,c). This further confirms that SS has a retarding effect on AAS paste at high Ms.

According to Figure 8, Figure 9 and Figure 10, it can be seen that the shear stress increases for all samples as time elapses. More hydration products are formed, leading to the higher shear stress of AASS and AASF pastes. The increase in the w/b ratio dramatically reduces the shear stress of the paste, which is attributed to the additional water content at high w/b ratio. The results are in agreement with the fluidity (Figure 4 and Figure 5) and setting time (Figure 7) results. The structural packing of AASS and AASF pastes gradually slows down with increasing w/b ratio. The higher water content reduces the alkalinity of activator, which slows down the dissolution rate of particles and thus results in slower structure formation. Furthermore, the porous microstructure is formed as a result of the higher water content. A larger number of products and longer times are required to build up a dense network structure. Therefore, the shear stresses of AASS and AASF pastes with w/b ratio of 0.5 are still low even at 120 min (Figure 10). 

Table 3 and Table 4 depict the rheological parameters obtained by fitting rheological curves with the H-B rheological model when the w/b ratios are 0.4 and 0.5, respectively. The yield stresses of all samples increase with time at the two studied w/b ratios. As seen from Table 3, the addition of SS or FA reduces the yield stress and plastic viscosity of the AAS paste. The reduction effect of FA on the yield stress and plastic viscosity of AAS paste is apparently higher than that of SS. This is because the spherical particles of FA are relatively smooth, while the SS particles are irregular and have a certain amount of RO phase. Raising the Ms evidently elevates the yield stress and plastic viscosity of AASS and AASF pastes. It is due to the initial floc formation and the high density of activator. The silicate concentration is not sufficient to contribute to the formation of primary C(N)-A-S-H gels at a low Ms, while the primary C(N)-A-S-H gel is generated at a high Ms [49]. Meanwhile, the presence of hydroxyl groups and silicates in the sodium silicate solution can facilitate the condensation reaction [50]. However, the opposite conclusion was obtained by Sun et al. [13,38], who found that a higher Ms led to a lower initial yield stress of the AAS paste. This is because the researchers only studied the rheological properties at low Ms (0.25 and 0.5 Ms), and they confirmed that the reduced solid concentration and the fluidizing effect of sodium silicate solution at 0.5 Ms decreased the initial yield stress. The increase in viscosity is due to the strong hydrogen bonding and a large number of colloidal particles in the sodium silicate solution at high Ms [46]. 

As shown in Table 4, the addition of SS and FA also reduces the yield stress and plastic viscosity of the AAS paste at 0.5 Ms when the w/b ratio is 0.5, but the reduction ratio is lower compared to that at w/b ratio of 0.4. When the Ms is 1.0, the incorporation of SS or FA slightly reduces the yield stress of AAS paste at 5 min but significantly reduces the yield stresses of AAS paste at 60 and 120 min. Note that the addition of SS or FA distinctly reduces the yield stress and plastic viscosity of the AAS paste at 1.5 Ms and w/b ratio of 0.5. It is indicated that the incorporation of SS or FA can obviously improve the rheological properties of AAS paste prepared with high Ms and high w/b ratio. In addition, the improving effect of SS on the rheological properties of AAS paste is more significant than that of FA at high Ms. The yield stress and plastic viscosity of AASS and AASF pastes also increase with increasing Ms at w/b ratio of 0.5, which is the same as that at w/b ratio of 0.4. Note that the initial fluidity of AAS paste and AASF paste increase with increasing Ms (Figure 4 and Figure 5), but the initial yield stress shows the opposite trend (Table 3 and Table 4). The fluidity indicates the flow ability of the paste, while the yield stress indicates the maximum stress that prevents plastic deformation of the paste. Thus, there is not a one-to-one correspondence between fluidity and yield stress.

Table 3 and Table 4 indicate that the increase in the w/b ratio significantly reduces the yield stress and plastic viscosity of AAS paste. The findings are in accordance with the fluidity results (Figure 4 and Figure 5). The increase in the w/b ratio increases the free water content in the paste and reduces the yield stress and plastic viscosity of AASS and AASF pastes.

## 4. Conclusions

Many researchers use FA to improve the workability and prolong the setting time of AAS paste. By comparing the effects of SS and FA on the hydration kinetics and rheological properties of AAS paste, the feasibility of using SS to improve the early properties of AAS paste was investigated in this study. Specific conclusions are as follows:

(1) SS shortens the induction period of AAS at low Ms yet prolongs the induction period at high Ms. The second exothermic peak values of AAS are also reduced by adding SS. The incorporation of SS increases the cumulative hydration heat of AAS at 0.5 Ms but decreases the cumulative hydration heat of AAS at 1.0 Ms and has limited influence on the cumulative hydration heat of AAS at 1.5 Ms. However, FA prolongs the induction period, reduces the exothermic peak value and decreases the cumulative hydration heat of AAS at the three studied Ms. 

(2) SS increases the fluidity of the AAS paste and increases the fluidity loss rate at low Ms, while the contrary law is obtained at high Ms. FA increases the fluidity of the AAS paste at 3 Ms. Increasing the w/b ratio increases the fluidity and reduces the fluidity loss rate of AAS paste containing SS or FA.

(3) The incorporation of SS shortens the setting time of the AAS paste at low Ms but prolongs the setting time at high Ms. The incorporation of FA prolongs the setting time of the AAS paste. The effect of SS on prolonging the setting time of AAS paste is enhanced with an increasing Ms, whereas the prolonging effect of FA is reduced. Increasing the w/b ratio increases the setting time.

(4) Either SS or FA reduces the yield stress and plastic viscosity of the AAS paste. SS or FA obviously improves the rheological properties of AAS paste at high Ms and high w/b ratio. The improvement effect of SS on the rheological properties of AAS paste is more significant than that of FA at high Ms. The yield stress and plastic viscosity of AAS paste containing SS or FA increase with the increasing Ms and decrease with the increasing w/b ratio. 

(5) SS and slag both belong to the iron and steel industry. SS can significantly improve the early properties of AAS paste at a high Ms. SS can replace FA to produce an AAS composite binder.

## Figures and Tables

**Table 1:** Chemical compositions of slag, FA and SS (w_t_/%).

Chemical Compositions	SiO_2_	Al_2_O_3_	Fe_2_O_3_	CaO	MgO	SO_3_	Na_2_O_eq_	LOI	Others
Slag	35.55	15.36	0.45	33.94	11.16	1.95	0.63	0.70	0.96
FA	57.60	21.90	7.70	3.87	1.68	0.41	4.05	0.43	2.79
SS	12.77	2.12	23.49	49.17	3.54	0.23	0.45	1.86	8.23

Na_2_O_eq_ = Na_2_O + 0.658K_2_O.

**Table 2:** Mix proportions of the pastes (wt/%).

Sample	Slag	SS	FA	Ms	w/b Ratio
GS1(GS1′)	100	0	0	0.5	0.4(0.5)
GS2(GS2′)	100	0	0	1.0	
GS3(GS3′)	100	0	0	1.5	
SS1(SS1′)	50	50	0	0.5	
SS2(SS2′)	50	50	0	1.0	
SS3(SS3′)	50	50	0	1.5	
FA1(FA1′)	50	0	50	0.5	
FA2(FA2′)	50	0	50	1.0	
FA3(FA3′)	50	0	50	1.5	

**Table 3:** Yield stress and plastic viscosity at w/b ratio of 0.4.

Sample	Hydration Time/(min)					
	5		60		120	
	*τ*_0_/(Pa)	*μ*/(Pa·s)	*τ*_0_/(Pa)	*μ*/(Pa·s)	*τ*_0_/(Pa)	*μ*/(Pa·s)
GS1	33.34 ± 1.47	1.26 ± 0.03	34.07 ± 1.2	1.56 ± 0.58	40.26 ± 1.56	1.428 ± 0.05
FA1	7.98 ± 0.32	0.65 ± 0.02	8.43 ± 0.21	0.70 ± 0.02	10.37 ± 0.21	0.73 ± 0.02
SS1	19.47 ± 0.86	0.67 ± 0.02	28.67 ± 1.02	0.71 ± 0.02	40.26 ± 1.52	0.66 ± 0.02
GS2	123.11 ± 6.02	1.42 ± 0.05	-	-	-	-
FA2	10.05 ± 0.42	0.75 ± 0.02	54.88 ± 1.62	1.25 ± 0.03	49.97 ± 1.68	1.24 ± 0.04
SS2	20.20 ± 0.88	0.64 ± 0.02	37.64 ± 1.23	0.95 ± 0.02	42.29 ± 1.67	1.00 ± 0.03
GS3	227.12 ± 8.23	1.31 ± 0.04	-	-	-	-
FA3	18.24 ± 0.85	0.67 ± 0.02	-	-	-	-
SS3	111.09 ± 4.86	0.67 ± 0.02	229.97 ± 8.68	1.54 ± 0.04	223.76 ± 9.86	3.11 ± 0.10

**Table 4:** Yield stress and plastic viscosity at w/b ratio of 0.5.

Sample	Hydration Time/(min)					
	5		60		120	
	*τ*_0_/(Pa)	*μ*/(Pa·s)	*τ*_0_/(Pa)	*μ*/(Pa·s)	*τ*_0_/(Pa)	*μ*/(Pa·s)
GS1’	4.31 ± 0.14	0.39 ± 0.01	7.52 ± 0.23	0.45 ± 0.01	8.13 ± 0.26	0.44 ± 0.01
FA1’	1.59 ± 0.05	0.15 ± 0.005	3.99 ± 0.12	0.14 ± 0.006	5.06 ± 0.22	0.15 ± 0.006
SS1’	2.59 ± 0.88	0.19 ± 0.006	7.21 ± 0.23	0.23 ± 0.01	11.57 ± 0.36	0.27 ± 0.01
GS2’	4.70 ± 0.12	0.51 ± 0.01	30.64 ± 1.22	1.66 ± 0.06	38.21 ± 1.05	1.89 ± 0.06
FA2’	1.70 ± 0.06	0.16 ± 0.006	8.85 ± 0.24	0.92 ± 0.03	9.04 ± 0.28	1.14 ± 0.03
SS2’	2.87 ± 0.11	0.24 ± 0.01	8.80 ± 0.26	0.53 ± 0.01	12.36 ± 0.42	0.59 ± 0.01
GS3’	19.14 ± 0.82	0.37 ± 0.01	-	-	-	-
FA3’	3.32 ± 0.12	0.16 ± 0.006	75.99 ± 2.68	1.02 ± 0.03	79.97 ± 2.52	1.06 ± 0.03
SS3’	3.92 ± 0.12	0.28 ± 0.01	22.01 ± 0.89	1.04 ± 0.03	25.25 ± 0.86	1.23 ± 0.04

## Data Availability

Data are contained within the article.
